# Does size really matter?

**DOI:** 10.7554/eLife.64483

**Published:** 2020-12-08

**Authors:** David G Heckel

**Affiliations:** Department of Entomology, Max Planck Institute for Chemical EcologyJenaGermany

**Keywords:** acari, miniaturization, genome reduction, reverse transcriptase-mediated intron loss, proboscipedia, horizontal gene transfer, Other

## Abstract

Analysis of the smallest known arthropod genome reveals a mechanism for genome reduction that appears to be driven by a specialized ecological interaction with plants.

**Related research article** Greenhalgh R, Dermauw W, Glas JJ, Rombauts S, Wybouw N, Thomas J, Alba JM, Pritham EJ, Legarrea S, Feyereisen R, Van de Peer Y, Van Leeuwen T, Clark RM, Kant MR. 2020. Genome streamlining in a minute herbivore that manipulates its host plant. *eLife* **9**:e56689. doi: 10.7554/eLife.56689

When it comes to animals, the saying ‘the bigger the better’ does not always hold true. Being small comes with some advantages, such as needing fewer resources or having more opportunities to hide or escape from predators. Take, for example, the tomato russet mite *Aculops lycopersici*, which is a pest that can cause serious damage to tomatoes and other related plants (including potatoes, tobacco and various peppers), even though it is among the tiniest animals on Earth and smaller than some single-celled organisms.

The tomato russet mite feeds on the outer epidermal cells of plant leaves by piercing the cell wall, secreting proteins and other compounds into the cell, and then sucking out the contents (Figure 1). Many plants rely on the jasmonic acid pathway to turn on their defenses against mites and other herbivores (Howe and Jander, 2008), but *Aculops* and other tomato-feeding mites can inhibit this pathway, although the mechanisms they use to do this remain unknown (Schimmel et al., 2018).

**Figure 1.**
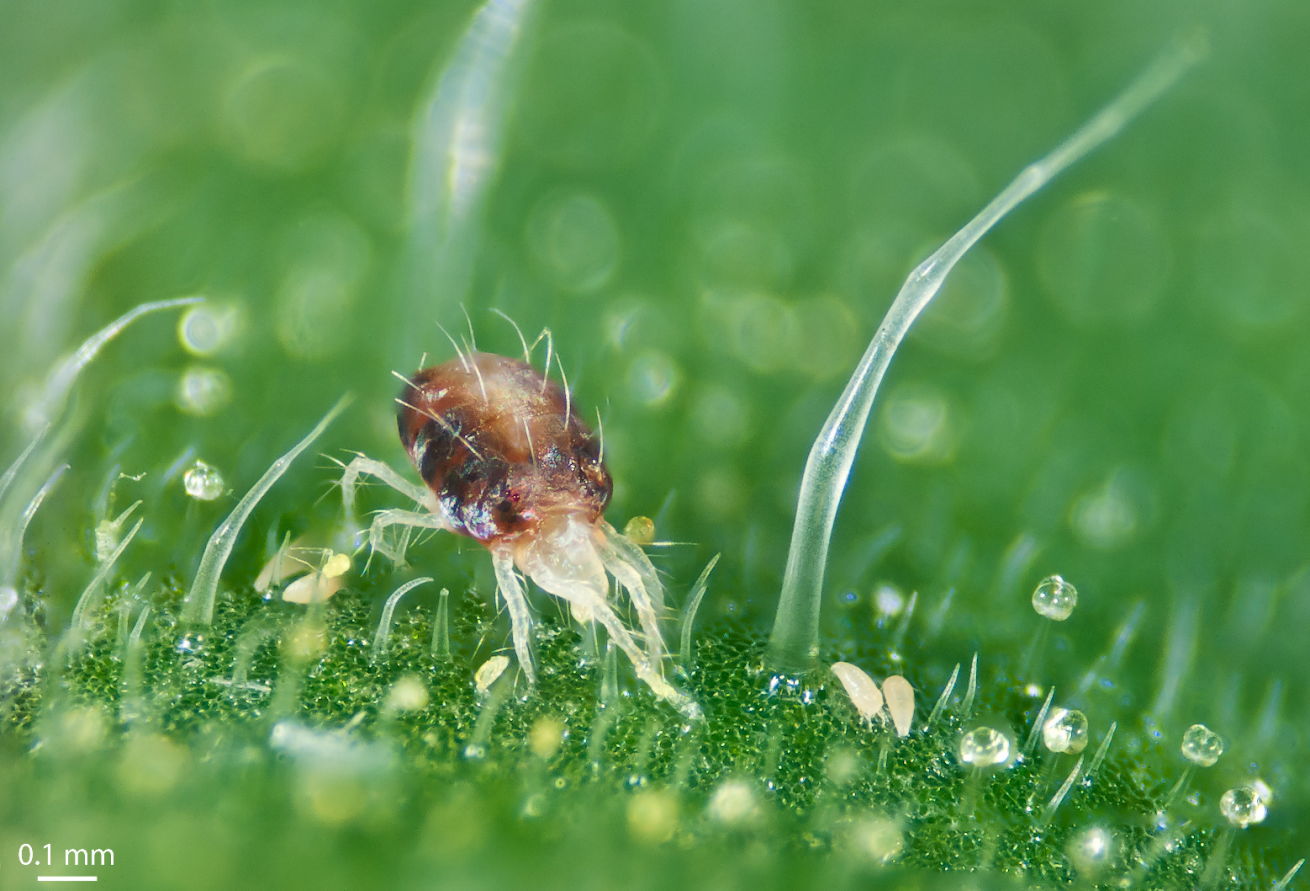
Mites on a leaf. A spider mite (brown, left) towers above two russet mites (white, right) among the trichomes of a tomato leaf. Both species can suppress the signaling pathways used by the plant to upregulate anti-herbivore defenses, but it remains unclear how tomato russet mites do this.

Image Credit: Jan van Arkel (IBED, University of Amsterdam) from Glas et al., 2014 (CC BY 4.0).

Comparative genomics of chelicerates – a large group of arthropods to which the tomato russet mite belongs – has already revealed significant differences to insects and could hold the clue to why these mites are such successful crop pests. Now, in eLife, Merijn Kant (University of Amsterdam), Richard Clark (University of Utah) and colleagues – including Robert Greenhalgh (University of Utah) and Wannes Dermauw (Ghent University) as joint first authors – report new insights on mite genomics (Greenhalgh et al., 2020).

The researchers sequenced the genome of *Aculops* mites and discovered that they have the smallest known arthropod genome to date,containing just 32.5 million bases. This was attained by an extreme reduction in both DNA content and gene number, especially in gene families involved in clearing toxins and sensing chemicals. Moreover, several transcription factors (proteins that help turn on and off genes) found in almost every other eukaryote were missing from the russet mite genome: this is puzzling because the mite is free-living, with no known symbiont that might complement its deficient gene repertoire.

The extent of DNA loss points to aggressive mechanisms for trimming down the genome. The average space between genes was about 540 base pairs, so that the number of base pairs between genes was roughly equal to the number coding for proteins. Any mechanism that has evolved to remove transposable elements (DNA sequences that can change their position within the genome) could achieve this, and also be responsible for the gene loss. Indeed, transposable elements account for less than 2% of the *Aculops* genome.

Moreover, there was a massive loss of non-coding DNA within genes: over 80% did not have any introns –regions of DNA that interrupt the coding sequence and are ‘spliced out’ before proteins are made. Even intron positions that are highly conserved across related species were absent in the tomato russet mite. For the other 20% of genes, most had retained their introns at the 5' end, but lost them at the 3' end – a pattern that has also been observed in other intron-poor organisms (Mourier and Jeffares, 2003; Roy and Gilbert, 2005).

The mRNA from which all the introns have been spliced out can serve as the template for an enzyme called reverse transcriptase, which makes single strands of DNA that are complementary to the mRNA. This process starts from the 3' end, and the resulting DNA can be integrated into the genome at the site of the original gene, replacing the original 3' end (which had introns) with a new 3' end (which has no introns). The 5' end (and its intron) remains intact. This mechanism would seamlessly ‘erase’ some introns (replacing the genomic DNA with intron-free sequence), rather than ‘excising’ them (physically cutting them out of the genomic DNA), although Greenhalgh et al. did find a few instances of imprecise excision. The enzymes responsible for these manipulations belong to the toolkit of retrotransposons, elements that can move by copying RNA into DNA, but they have yet to be identified in the russet mite.

Although the mechanisms of genome reduction can be envisioned, the evolutionary driving force behind them remains an enigma. Plant-parasitizing mites seem to have created their own niche among herbivores by manipulating plant biochemistry in ways where a small body size is an advantage. Shielded by leaf hairs and crevices, these tiny leaf feeders remain well hidden from most predators. Some mite species even hormonally manipulate plant structures into galls that encase them. The epidermal cells in plants are also relatively poor in nutrients, putting a premium on small size and high efficiency.

Paradoxically, the extreme genome reduction of *Aculops lycopersici* runs counter to adaptations of most insect herbivores studied to date, which usually show great expansions in gene families relevant for sensing chemicals and destroying toxins. How this mite can manipulate a plant’s defense mechanisms continues to remain a mystery. Comparisons to other tomato-feeding mites showed few similarities in the composition of saliva proteins, which are presumed to inactivate the plant's defensive signaling pathways.

So, what scenarios could explain a tendency to minimize the genome, and could they really lead to better adaptations of the parasite? The fitness cost of gene loss might not immediately be balanced by a reduced size, but a short generation time of four days would facilitate a rapid evolutionary adjustment to compensate. Since infestations may start with one or a few dispersing individuals but then rapidly explode to huge population sizes, random genetic drift could further accelerate the process. Can (small) size matter so much to fitness to sustain the continuous and gradual erosion of DNA that ultimately shaped the russet mite of the 21^st^ century? Unraveling the evolutionary dynamics of this process could be the greatest benefit of a comparative study of parasitic mites and their relatives.
